# Characterization of Urinary *N*-Acetyltaurine as a Biomarker of Hyperacetatemia in Mice

**DOI:** 10.3390/metabo14060322

**Published:** 2024-06-07

**Authors:** Qingqing Mao, Xiaolei Shi, Yiwei Ma, Yuwei Lu, Chi Chen

**Affiliations:** Department of Food Science and Nutrition, University of Minnesota, 1334 Eckles Ave., St. Paul, MN 55108, USA; maoxx113@umn.edu (Q.M.); shixx121@umn.edu (X.S.); maxxx792@umn.edu (Y.M.); luxxx572@umn.edu (Y.L.)

**Keywords:** *N*-acetyltaurine, acetate, hyperacetatemia, metabolite biomarker, metabolomics

## Abstract

Acetate is an important metabolite in metabolic fluxes. Its presence in biological entities originates from both exogenous inputs and endogenous metabolism. Because the change in blood acetate level has been associated with both beneficial and adverse health outcomes, blood acetate analysis has been used to monitor the systemic status of acetate turnover. The present study examined the use of urinary *N*-acetyltaurine (NAT) as a marker to reflect the hyperacetatemic status of mice from exogenous inputs and endogenous metabolism, including triacetin dosing, ethanol dosing, and streptozotocin-induced diabetes. The results showed that triacetin dosing increased serum acetate and urinary NAT but not other *N*-acetylated amino acids in urine. The co-occurrences of increased serum acetate and elevated urinary NAT were also observed in both ethanol dosing and streptozotocin-induced diabetes. Furthermore, the renal cortex was determined as an active site for NAT synthesis. Overall, urinary NAT behaved as an effective marker of hyperacetatemia in three experimental mouse models, warranting further investigation into its application in humans.

## 1. Introduction

Acetate is a ubiquitous metabolite in nature. Its presence inside the human body originates from both exogenous and endogenous sources [1]. Exogenously, acetate could be directly obtained via dietary intake of acetate-containing foods and beverages, such as apple cider vinegar [2] and fermented foods [3], or medical procedures, such as acetate supplementation and the infusion of acetate-containing buffers [4]. Alcohol intake is an indirect source of acetate in humans as acetate is the end product of the ethanol–acetaldehyde–acetate metabolic cascade in alcohol metabolism [5]. Endogenously, acetate is produced via multiple pathways. In the liver, acetate can stem from acetyl-CoA hydrolysis by cytoplasmic acylthioesterase-12 (ACOT12) to free coenzyme A for other metabolic activities [6]. For the cells under excessive nutritional supply, acetyl-CoA-independent metabolic activities of keto acid dehydrogenases are capable of converting pyruvate to acetate de novo through either thiamine- or reactive oxygen species (ROS)-dependent manners [7]. Acetate could also be released from the deacetylation reactions, such as from acetylated proteins [8]. In addition, gut microbiota produces acetate as a major fermentation product, which is then extensively absorbed and uptaken by the liver [9].

The disposition and functions of acetate inside the body have been extensively investigated [10]. Acetate is transported into cells by both passive diffusion and monocarboxylate transporters-mediated facilitated diffusion [11]. Inside the cell, through the catalysis of acetyl-CoA synthetases [12], the direct usage of acetate is to produce acetyl-CoA, which is an essential intermediate in both catabolic and anabolic metabolism as well as the cofactor for the acetylation of proteins, nucleotides, and other amine-containing metabolites [13]. Meanwhile, acetate is a ligand of free fatty acid receptors 2 (FFAR2, also known as GPR43), which is a mediator for pancreatic β-cell function [14] and immune responses [15]. Acetate plays versatile roles in human health and disease through these metabolic and regulatory functions. In many cases, acetate is considered beneficial as the acetate from gut microbial fermentation of dietary fiber or from dietary supplementation, such as apple cider vinegar, has been shown to lower blood cholesterol, enhance insulin sensitivity, decrease body weight, expedite fatty acid oxidation, and reduce pro-inflammatory cytokines [2,16,17,18,19]. On the other hand, the associations of acetate with adverse health events have also been reported [9]. For example, increased plasma acetate levels after feeding rats with high-fat and high-calorie diets correlated with hyperinsulinemia, increased fat deposition, and weight gain [20]. Higher blood acetate level was associated with increased cancer risk [21], partially due to its usage as an energy source and a substrate for the lipogenesis and proliferation of cancer cells, especially the aggressive types [22,23]. Additionally, acetate, when used in the buffer of hemodialysis, has been implicated in the development of hypotension and hypoxemia through its vasodilatory effect [24]. Considering these double-edged effects of acetate, monitoring the status of circulating acetate levels carries clear values in both research and clinical applications. Currently, acetate levels are commonly monitored using blood samples as well as selected biological samples, such as the liver and fecal samples. Nonetheless, such biological specimens may not always be accessible due to invasive and inconvenient collection procedures. Hereby, we propose using urinary *N*-acetyltaurine (NAT) as a biomarker to reflect the systematic acetate level.

NAT was initially discovered in mice as an ethanol metabolite in our ethanol-feeding study, in which the kidney was identified as an active organ conducting the reaction between acetate and taurine to form NAT [25]. Subsequent studies confirmed NAT as an ethanol metabolite in human urine [26] and further showed that urinary NAT was increased by endurance exercises [27,28]. Considering the fact that acetate is the direct precursor of NAT and taurine is an abundant free amino acid, the potential utilization of urinary NAT as an alternative marker of hyperacetatemia from exogenous inputs or endogenous metabolism has been proposed but not investigated [25]. This study examined the correlations between serum acetate and urinary NAT in three animal models of hyperacetatemia, including direct acetate intake by triacetin administration, indirect input from ethanol feeding, and endogenous acetate formation under diabetes. In addition, the site of NAT synthesis in the kidney was examined by comparing the NAT synthesizing activity between the renal cortex and medulla samples in vitro.

## 2. Materials and Methods

### 2.1. Chemicals and Reagents

The chemicals and reagents used in sample preparation, LC–MS analysis, structural confirmation, and quantification are listed in Table S1. NAT was synthesized and authenticated in-house in a previous study [25].

### 2.2. Animals, Experimental Design, and Sample Collection

Animal care and experimental procedures were approved by the University of Minnesota Institutional Animal Care and Use Committee (IACUC). Male C57BL/6 mice, 8–12-weeks old, were purchased from Charles River Lab (Wilmington, MA, USA). All mice were housed in the University of Minnesota animal facility at a constant temperature of 21 °C under a 12 h light–dark cycle, with *ad libitum* access to water and feed. The mice were first acclimated to the normal chow for 7 days before corresponding dosing experiments. Blood samples were collected via submandibular bleeding and centrifuged at 3000× *g* for 20 min to obtain serum samples. The 24-h urine samples were collected after housing individual mice in metabolic cages for 24 h. All samples were stored at −80 °C prior to the analysis.

*Direct acetate intake through triacetin dosing.* For triacetin feeding (IACUC protocol 2208-40322A, approved on 2 September 2022), two groups of male C57BL/6 mice were gavaged with 40% (*v*/*v* in water) glycerol at the dose of 2.52 g/kg body weight (n = 5) and triacetin at the dose of 5.8 g/kg body weight (n = 4), respectively. The selected dose of triacetin was known to increase the acetate level in mice brains within 1–2 h of oral gavage [29]. The selected dose of glycerol was equivalent to the molar quantity of the glycerol moiety in dosed triacetin. Serum samples were collected at 2 h after dosing. Urine samples were collected for 24 h after dosing.

*Indirect acetate input from ethanol (EtOH) dosing.* For ethanol feeding (IACUC protocol 1211A24284, approved on December 13, 2012), two groups of male C57BL/6 mice were fed the control semi-solid dextrose diet (n = 4) and a modified semi-solid diet formulated based on the Lieber–DeCarli liquid ethanol diet (n = 8), respectively, for 15 days. The details on the preparation and feeding of semi-solid diets were described previously [25]. Ethanol concentration in the diet started at 2.2% (*v*/*v*), increased to 4.5% on day 4, and 6.7% on day 9. Urine and serum samples were collected on day 0 (D0) before feeding and day 15 (D15) of feeding, respectively.

*Endogenous acetate input from streptozotocin (STZ) dosing.* Diabetes was induced by intraperitoneal injection of streptozotocin (STZ) at the dose of 180 mg/kg (IACUC protocol 1211A24284, approved on December 13, 2012). Two groups of male C57BL/6 mice (n = 4 per group) were dosed with STZ alone and STZ with 2% (*w*/*v*) taurine in drinking water, respectively. The selected dose of taurine supplementation has been reported to improve the glucose tolerance of Wistar rats under a high-fructose diet [30]. Individual mice were housed in metabolic cages with urine and serum samples collected on day 0 (D0) before STZ dosing and on day 6 (D6) of the treatment, respectively. The development of diabetes was confirmed by measuring blood glucose using a glucometer.

### 2.3. Sample Preparation and Chemical Derivatization for LC-MS Analysis

For quantifying urinary NAT, 100 µL of urine samples were quenched with 400 µL of 50% aqueous acetonitrile containing 5 µM sulfadimethoxine as the internal standard for protein precipitation. To analyze creatinine, 10 µL urine samples were diluted with 1000 µL of 50% aqueous acetonitrile containing 5 µM sulfadimethoxine as the internal standard. These urine samples were centrifuged at 13,000× *g* for 10 min, and their supernatants were submitted for LC–MS analysis. Serum samples were quenched with 2 volumes of acetonitrile containing 5 µM sulfadimethoxine as the internal standard, then centrifuged to acquire the supernatant.

For detecting and quantifying taurine, the serum and urine samples were derivatized by dansyl chloride (DC) prior to LC–MS analysis. In brief, 5 μL of quenched urine, serum, or taurine standard was mixed with 5 μL of 50 μM deuterated *d*_5_-tryptophan (internal standard), 50 μL of 10 mM sodium carbonate, and 100 μL of DC (3 mg/mL in acetone). The mixture was incubated at 60 °C for 15 min and then centrifuged at 13,000× *g* for 10 min. Then, the supernatant was transferred to an HPLC vial for LC–MS analysis.

For detecting and quantifying acetate, the serum and urine samples were derivatized by 2-hydrazinoquinoline (HQ), following an established protocol [31]. In brief, 2 µL of quenched sample was mixed with 100 µL of freshly prepared acetonitrile solution containing 1 mM 2-2′-dipyridyl disulfide (DPDS), 1 mM triphenylphosphine (TPP), 1 mM HQ, and 20.8 µM deuterated *d*_4_-acetate (internal standard). After incubation at 60 °C for 30 min, chilled on ice, the reaction was stopped by mixing with 100 µL of ice-cold water. After centrifuging at 13,000× *g* for 15 min, the supernatant was transferred to an HPLC vial for LC-MS analysis.

### 2.4. In Vitro Incubation of Renal Tissues for NAT Synthesis

To determine the site of NAT synthesis inside the kidney, cow kidneys were selected for sampling renal cortex and medulla tissues due to their large size. Fresh cow kidneys were obtained from grass-fed Angus beef cattle at the time of slaughtering. The kidney lobes were sliced perpendicular to their longitudinal axis to expose the medulla and cortex tissues. After the collection, the tissue samples were stored at −80 °C prior to the analysis. The tissue homogenates were prepared by homogenization in a buffer containing 320 mM sucrose, 50 mM phosphate buffered saline (PBS) solution, 1 mM EDTA, and protease inhibitor. The mixture was centrifugated at 600× *g* for 10 min to remove the nuclear pellet. The in vitro reaction was conducted by incubating the homogenate with 20 mM taurine and 2.5 mM acetate in a phosphate-buffered saline solution at 37 °C for 30 min. The reaction was terminated by adding an equal volume of acetonitrile. The yield of NAT was quantified via the LC–MS analysis of the reaction mix.

### 2.5. LC-MS Analysis

An aliquot of 2 µL sample from the abovementioned preparation procedures was injected into a Waters Acquity Ultra-Performance Liquid Chromatography (UPLC) system (Milford, MA, USA) and separated in a column. The eluant from the LC was then introduced into a Waters Xevo-G2-S or Synapt-G2-Si quadrupole time-of-flight mass spectrometer (QTOF-MS) for accurate mass measurement and ion counting. Nitrogen was used as both cone gas (50 L/h) and desolvation gas (600 L/h). The mass analyzer was calibrated to detect ions within the range *m/z* 50–1200 and was monitored by intermittent lock mass injection. The structures of interested metabolites were analyzed by tandem MS (MS/MS) fragmentation with a collision energy ramp of 10–50 eV. The column, mobile phases, mass detection mode, capillary and cone voltages, and lock mass used for different metabolites are included in Table S2.

Mass chromatograms and mass spectral data were acquired and processed by MassLynx^TM^ software V4.2 (Waters) in centroided format. Target compounds were quantified by accurate mass-based chromatograms. Standard curves of creatinine, NAT, *N*-acetyl-glutamate (NA-Glu), *N*-acetyl-glutamine (NA-Gln), *N*-acetyl-isoleucine (NA-Ile), *N*-acetyl-leucine (NA-Leu), *N*-acetyl-phenolalanine (NA-Phe), *N*-acetyl-tyrosine (NA-Tyr), and taurine (5 to 1000 μm) were prepared. The concentration of target compounds in the samples was determined by fitting the peak area (normalized by corresponding internal standards) with the standard curves via Quanlynx^TM^ (Waters). The extracted ion chromatograms (EICs) of targeted analytes are shown in Figure S1.

### 2.6. Untargeted Multivariate Data Analysis

The LC–MS data were analyzed via MarkerLynx^TM^ software V4.2 (Waters). The chromatographic and spectral data underwent centroiding, deisotoping, filtering, peak recognition, and integration. Then, a multivariate data matrix containing sample identity, ion identity (retention time and *m/z*), and ion abundance was generated and visualized via SIMCA^TM^ software version 13.0 (Sartorius, Göttingen, Germany). The intensity of each single ion was normalized to the total ion counts, and the total ion intensity was set arbitrarily at 10,000. The processed data matrix was further transformed by Pareto scaling and then analyzed using principal components analysis (PCA). Major latent variables in the data matrix were described in a scores scatter plot of the multivariate model. The chemical identity of ions of interest was determined by accurate mass measurement, database search (HMDB www.hmdb.ca, accessed on 4 March 2024), and elemental composition analysis. The data matrix generated from MarkerLynx^TM^ V4.2 (Waters) analysis was also log-transformed, Pareto scaled, and then processed by Metaboanalyst 6.0 (https://www.metaboanalyst.ca/, accessed on 11 March 2024) to construct the volcano plot.

### 2.7. Statistical Analysis

The concentrations or relative abundances of targeted metabolites were expressed as the mean ± standard deviation (SD). The statistical analysis was conducted by two-way ANOVA with Fisher’s LSD tests on the rank-transformed data (RT-1, ranking of all the data together) or non-paired *t*-test via Graphpad Prism 5^TM^ (Dotmatics, Boston, MA, USA). The difference was considered significant with the *p*-value < 0.05.

## 3. Results

### 3.1. Influences of Triacetin Dosing on Urinary NAT and Other Urinary Metabolites

**Figure 1:**
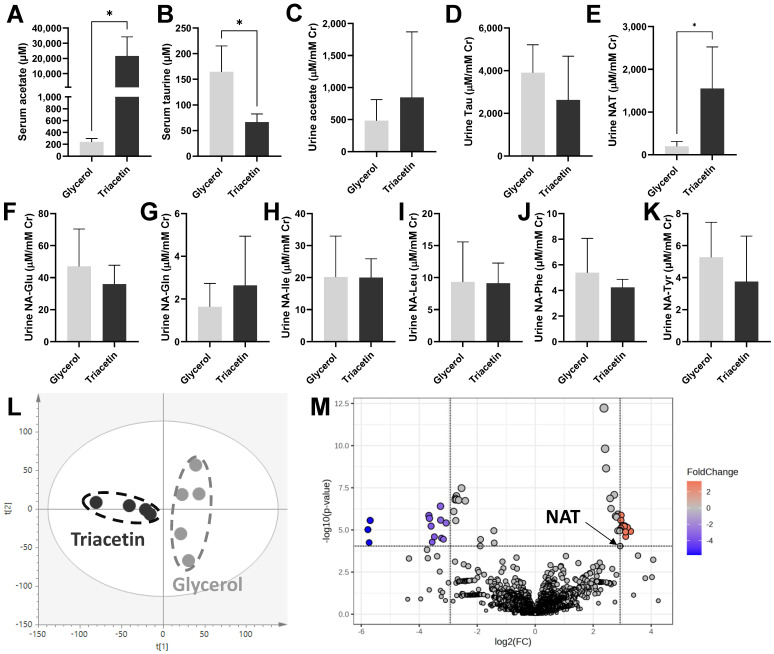
Concentrations of targeted serum and urine metabolites and the multivariate modeling of urine metabolome after triacetin dosing. Serum samples were collected 2 h post dosing. Urine samples were collected after housing mice individually in metabolic cages for 24 h post dosing (n = 5 in the glycerol group, n = 4 in the triacetin group). (**A**) serum acetate, (**B**) serum taurine, (**C**) urine acetate, (**D**) urine taurine, (**E**) urine NAT, (**F**) urine *N*-acetyl-glutamate (NA-Glu), (**G**) urine *N*-acetyl-glutamine (NA-Gln), (**H**) urine *N*-acetyl-isoleucine (NA-Ile), (**I**) urine *N*-acetyl-leucine (NA-Leu), (**J**) urine *N*-acetyl-phenylalanine (NA-Phe), (**K**) urine *N*-acetyl-tyrosine (NA-Tyr). * indicates *p*-value < 0.05 from non-paired t-test. (**L**) Scores plot of the PCA model on combined positive- and negative-mode data on the urine metabolome, in which t[1] and t[2] stand for the principal components 1 and 2, respectively. (**M**) Volcano plot of the urinary metabolites contributing to the differentiation between the triacetin- and glycerol-dosing groups. The fold-change (FC) and *p*-value of individual metabolites are the ratios of triacetin vs. glycerol control and their statistical significances, respectively. The metabolites with a greater FC and more significant *p*-value (red colored) than NAT (marked) were selected for further analysis (Table S3).

Direct dosing of acetate was achieved by gavaging triacetin, a triglyceride with three units of acetate, to mice. The triacetin treatment increased serum acetate drastically and also decreased serum taurine at 2 h of dosing compared to the glycerol control treatment (Figure 1A,B). In contrast, both acetate and taurine in the 24-h pooled urine were not affected by triacetin dosing (Figure 1C,D). Nevertheless, triacetin increased urinary NAT by more than six times (Figure 1E). To determine whether the observed increase of urinary NAT occurred to other acetylated amino acids, the concentrations of a group of *N*-acetyl amino acids (NAAAs) in urine were measured. The results showed that the concentrations of urinary *N*-acetyl-glutamate, *N*-acetyl-glutamine, *N*-acetyl-isoleucine, *N*-acetyl-leucine, *N*-acetyl-phenylalanine, and *N*-acetyl-tyrosine were comparable between the glycerol and triacetin groups (Figure 1F–K). To further examine the uniqueness of NAT as a responsive urinary metabolite of hyperacetatemia, unsupervised multivariate modeling on the urinary metabolome was constructed using the data from both positive- and negative-mode MS analysis of urine samples. In the scores plot of the constructed PCA model, a clear separation of the glycerol control and triacetin treatment was observed (Figure 1L). NAT was reconfirmed as a urinary metabolite contributing to the differentiation of two feeding groups in a volcano plot (Figure 1M). Other urinary metabolites with greater fold change (FC) and smaller *p*-values than NAT were further investigated for their structural identities by accurate mass-based elemental composition analysis, database search, and MSMS fragmentation (Table S3). No more acetylated metabolites were identified among them, except one negative ion with *m/z* = 309.0815 because of the neutral loss of the acetyl group (the loss of 42 from *m/z* 309 → 267) in its MSMS spectrum. However, this metabolite was absent in the control urine, indicating its identity as a specific metabolite of triacetin, not an endogenous metabolite. Overall, NAT was the sole identified *N*-acetylated endogenous metabolite in the urine that is responsive to the triacetin treatment.

### 3.2. Influences of Ethanol Dosing on Urinary NAT

**Figure 2:**
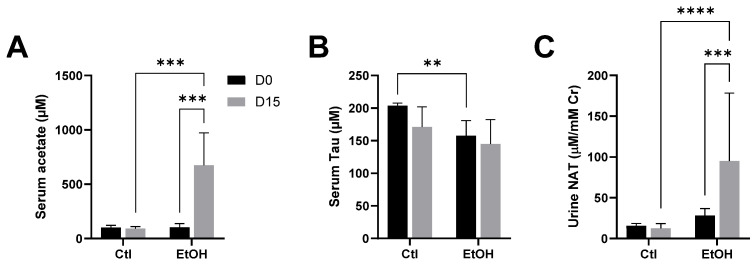
Serum acetate and taurine and urinary NAT after 15-day ethanol dosing. Serum and urine samples were collected from the control (Ctl) group (n = 4) and the ethanol (EtOH) feeding group (n = 8) on day 0 (D0) before feeding the semi-liquid diets and on day 15 (D15) of the feeding. (**A**) serum acetate, (**B**) serum taurine, (**C**) urine NAT. Two-way ANOVA tests were conducted on the rank-transformed data, followed by Fisher’s LSD tests to examine the differences within the same treatment across days and between two treatments on the same day (** *p* < 0.01, *** *p* < 0.001, **** *p* < 0.0001).

Ethanol feeding was performed to achieve indirect input of acetate since acetate is the main intermediate in ethanol metabolism. The results showed that ethanol feeding increased serum acetate but did not affect serum taurine (Figure 2A,B). In the meantime, ethanol increased urinary NAT excretion (Figure 2C).

### 3.3. Influences of STZ-Induced Diabetes and Taurine Supplementation on Urinary NAT

**Figure 3:**
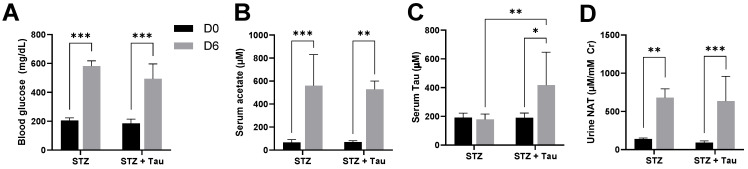
Blood glucose, serum acetate and taurine, and urinary NAT after STZ dosing and taurine supplementation. Serum and urine samples were collected from the STZ alone (STZ) treatment group (n = 4) and the SZT plus 2% (*w*/*v*) taurine in drinking water (STZ+Tau) treatment group on day 0 (D0) before STZ treatment and on day 15 (D15) after the treatment (n = 4 in each treatment group). (**A**) Blood glucose, (**B**) serum acetate, (**C**) serum taurine, (**D**) urinary NAT. Two-way ANOVA tests were conducted on the rank-transformed data, followed by Fisher’s LSD tests to examine the differences within the same group across days and between the two groups on each day (* *p* < 0.05, ** *p* < 0.01, *** *p* < 0.001).

In addition to the exogenous acetate from triacetin and ethanol dosing, the effect of endogenous metabolism-derived acetate on urinary NAT was examined in the STZ-induced diabetes mouse model. The influence of taurine supplementation on serum acetate and urinary NAT was further examined by the joint treatments of STZ injection and 2% taurine in drinking water. The results showed that STZ effectively induced hyperglycemia (Figure 3A) and increased serum acetate and urinary NAT (Figure 3B,D) but did not affect serum taurine (Figure 3C). On the other hand, taurine supplementation increased serum taurine (Figure 3C) but did not affect STZ-elicited effects on serum acetate (Figure 3B) and urinary NAT (Figure 3D).

### 3.4. Site of NAT Synthesis Site in the Kidney

**Figure 4:**
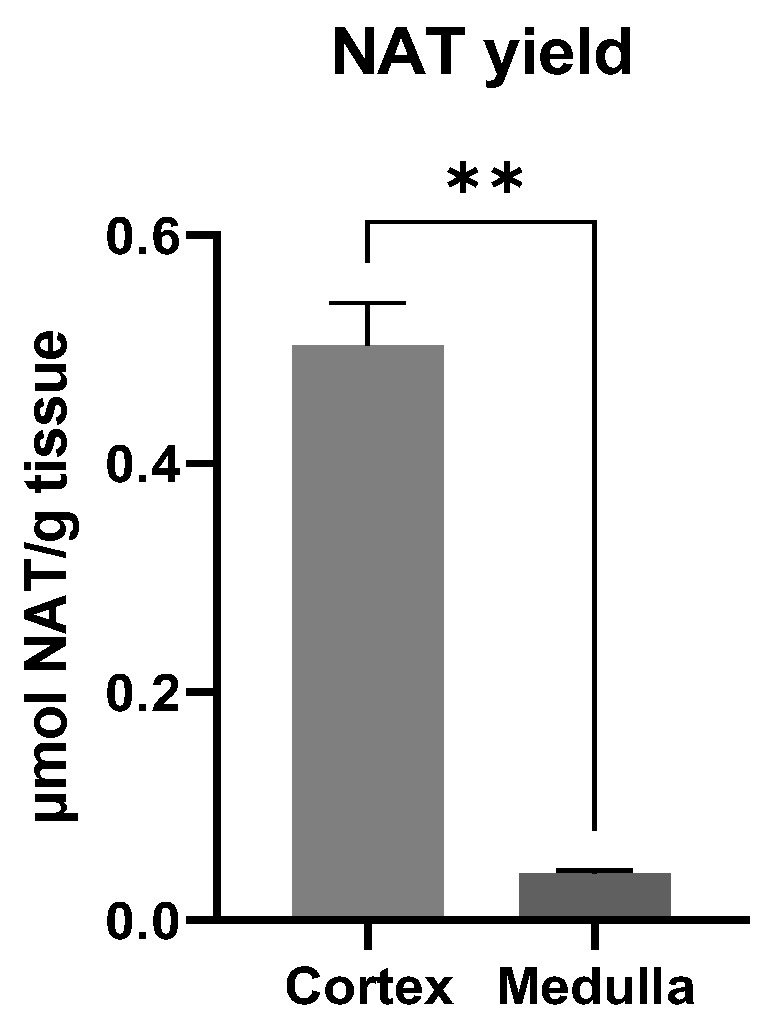
The yield of NAT synthesis from 30-min incubations of bovine renal cortex and medulla homogenates with acetate and taurine. ** indicates *p* < 0.01 from a non-paired t-test.

The kidney was previously identified as a more active organ than the liver and other organs/tissues for NAT synthesis in mice [25]. However, the site of NAT synthesis inside the mouse kidneys was not defined due to their small size. To address this question, bovine kidneys were used to harvest the renal cortex and medulla fractions. The results from the in vitro incubations showed that the NAT production from the cortex fraction was much greater than that of the medulla fraction (Figure 4), indicating that the renal cortex is an active site for the NAT synthesis from taurine and acetate.

## 4. Discussion

**Figure 5:**
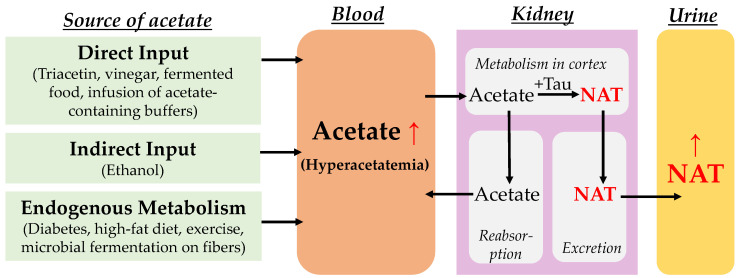
The sources of hyperacetatemia and the metabolic events leading to the increased urinary NAT. Blood acetate can be increased by diverse direct and indirect exogenous inputs as well as various endogenous metabolic events. In the kidney, after elevated acetate reacts with taurine to form NAT in the renal cortex, free acetate may undergo extensive reabsorption while NAT is readily excreted into urine, making urinary NAT, instead of urinary acetate, highly responsive to hyperacetatemia.

In this study, NAT was validated as a urinary metabolite marker of hyperacetatemia in three experimental mouse models, including direct dosing through triacetate, indirect input through ethanol metabolism, and endogenous production under diabetes (Figure 5). The biochemical events underlying NAT synthesis, including the sources of acetate and the biochemical properties of the reaction between acetate and taurine, as well as the advantages and values of NAT as a biomarker of metabolic status and disorders, are discussed as follows.

### 4.1. Current Knowledge of the Biochemical Properties of NAT Synthesis

NAT is not the only *N*-acetylated amino acid (NAAA) in urine, but it was the only one responsive to triacetin-induced hyperacetatemia in this study (Figure 1). The biosynthesis of NAAAs has been extensively studied since they have been widely characterized as the urinary markers of metabolic disorders, such as inborn errors of metabolism [32], neurodegenerative diseases [33], and kidney disease [34]. The formation of the NAAAs from proteinogenic amino acids, such as *N*-acetylglutamate and *N*-acetylphenylalanine, is mainly through the acetylation of respective amino acids by the transferases using acetyl-CoA as the substrate [35]. In contrast, the formation of NAT by kidney homogenates in the current study was through the direct reaction of taurine and acetate (Figure 4). This observation of direct taurine–acetate reaction is consistent with our previous investigation on the enzymatic kinetics of NAT synthesis, in which acetate was determined as a more favorite substrate for NAT synthesis than acetyl-CoA based on the facts that the taurine–acetate reaction had similar K_m_ value (affinity) but greater V_max_ value (capacity), in comparison with the taurine–acetyl-CoA reaction [25]. Together, the enzymatic analysis in our previous study and the incubations of renal fractions in the current study have defined the properties of the unknown enzyme responsible for NAT synthesis as a cytosolic metalloenzyme abundant in the kidney cortex that is capable of catalyzing the esterification reaction between taurine and acetate without the involvement of ATP and CoA [25]. Despite this knowledge, the exact identity of NAT synthase, its distribution, and its regulation remain to be determined.

In addition to acetate, taurine is a required substrate for NAT synthesis. Three known function- and distribution-related features of taurine are relevant to the NAT synthesis and the concentrations of its precursors in the three mouse models of hyperacetatemia in this study. Firstly, taurine, as a highly abundant free amino acid and also an intracellular osmolyte, is actively synthesized inside the body. Its concentration in many mammalian organs and tissues, including the kidney, ranges between 5 and 50 mM [36], which is much greater, by 2–3 orders of magnitude, than its blood concentrations, including the ones observed in this study. Therefore, the observed changes in serum taurine, including the decrease from triacetin treatment or the increase from 2% taurine supplement in the STZ-induced diabetes model, might have limited influence on the availability of intracellular taurine for the enzymatic performance in NAT synthesis, which may further explain the lack of increase in urinary NAT after the joint treatment of STZ and taurine (Figure 3D). Secondarily, taurine turnover in the kidney is adjustable since increased urinary excretion and increased renal uptake were observed under taurine overloading and taurine deficiency, respectively [37,38]. This robust regulation can effectively maintain the systematic taurine pool, hence providing adequate taurine substrate for NAT synthesis. Lastly, taurine is known for its antioxidant and cytoprotective functions, contributing to its known activities against ethanol-induced liver injury [39] and STZ-induced diabetes in rodent models [40]. It will be interesting to investigate whether NAT, a direct derivative of taurine, shares these beneficial activities of taurine in future studies.

### 4.2. Urinary NAT as a Marker of Hyperacetatemia: A Comparison with Serum and Urinary Acetate

The three mouse models of hyperacetatemia in this study reflected the hyperacetatemic scenarios that could occur to humans through intentional acetate consumption, alcohol overdose, and diabetes. Many more events could also elevate serum acetate in humans and animals, such as vinegar consumption and supplementation, high-fat diet, endurance exercise, gut microbial fermentation on the fiber, as well as pathophysiological events [3,20,27,28,41,42]. Measuring blood acetate concentration can definitely determine the status quo of acetate at the moment of sampling. However, it might not accurately reflect the overall status of acetate homeostasis since blood acetate concentration can change rapidly after acetate inputs. For example, post-prandial plasma acetate concentration in humans peaked at 30 min after breakfast and then decreased rapidly [43]. Additionally, the plasma concentration of acetate in human subjects after consuming 15 mL of apple cider vinegar peaked at 1 h and then returned to the baseline after 2 h of dosing [3]. Therefore, timely sampling or multiple samplings are needed to detect hyperacetatemia through blood acetate measurement. By contrast, urinary NAT has advantages for detecting hyperacetatemia based on the following considerations: (1) Urine is accumulative and covers a broader time window than blood; (2) for humans, urine collection is more compliant and less invasive than blood collection; and (3) detecting NAT is more convenient than detecting acetate, at least for the LC–MS analysis in the current study, where acetate detection required chemical derivatization, whereas NAT was directly detected.

As for urinary acetate, in contrast to the drastic increase of serum acetate, acetate in pooled urine samples from triacetin-treated mice did not increase. The lack of the change in urinary acetate, in this case, might be explained through acetate metabolism and renal functions in reabsorption. In the kidney, acetate is actively metabolized to bicarbonate, as shown by the usage of sodium acetate in dialysis, which leads to urine alkalinization instead of acidosis through the excretion of bicarbonate from acetate metabolism into urine due to compromised bicarbonate reabsorption function in the patients of renal diseases [42,44]. In addition, monocarboxylic acid transporters capable of acetate uptake are highly abundant in the renal proximal tubule, which may further prevent unnecessary leakage of acetate into urine [45,46]. However, when this mechanism of metabolism and reabsorption was either overwhelmed by acetate dosing or compromised by renal injury, the elevations of urinary acetate were also observed [47,48], leading to the usage of urinary acetate in the diagnosis of urinary tract infection [49] and ischemia-reperfusion injury after kidney transplantation [48]. Therefore, under normal renal function, urinary NAT has an advantage over urinary acetate in sensitivity for detecting hyperacetatemia.

In addition to its presence in mouse urine, NAT as a constitutive molecule of human urine has been established [26]. The elevation of its levels in human urine and blood has also been observed after endurance exercises [27,28]. Based on the results from the current study, more investigations are needed to establish it as a marker of hyperacetatemia in humans and determine its value in the monitoring of exogenous acetate intake, such as vinegar supplementation, and the diagnosis of metabolic disorders.

### 4.3. Limitations

In addition to confirming the increases of urinary NAT in three mouse models of hyperacetatemia, our data analysis also revealed the limitations of the current study. Firstly, relatively small numbers of animals used in each model might contribute to the observed large variance among individual animals within the same treatment group, which is reflected by the high standard deviations in some measured values. Secondly, to explore the correlation between urinary NAT and serum acetate, their values in three models of hyperacetatemia were processed by linear regression, showing a significant correlation (Figure S2A). However, this correlation was mainly caused by the values from the triacetin treatment since the exclusion of triacetin data made the correlation insignificant (Figure S2B). Such challenge of this correlation analysis can be partially attributed to the different properties between serum and urine because serum samples were collected at single time points while urine samples were accumulative. In future studies, a more reliable analysis of the correlation between urinary NAT and serum acetate may be achieved by measuring the dose–response of urinary NAT to different acetate inputs or by determining the area under the curve (AUC) of serum acetate during the period of urine collection for NAT analysis.

## 5. Conclusions

The current study examined the performance of urinary NAT as an indicator of elevated serum acetate in three mouse models of hyperacetatemia. Considering the importance of acetate in general metabolic fluxes and many pathophysiological events, the use of urinary NAT as a marker of hyperacetatemia could provide a convenient and effective venue for monitoring the status of acetate-related metabolism as well as for clinical diagnosis.

## Data Availability

The data presented in this study are available in the main article, the Supplementary Materials, and upon request.

## Supplementary Materials

The following are available online at https://www.mdpi.com/article/10.3390/metabo14060322/s1: Table S1: Reagents and chemicals used in sample preparation and LC–MS analysis. Table S2: LC–MS instrumental settings for data acquisition. Table S3: Information on selected triacetin-responsive urinary metabolites. Figure S1: Extracted ion chromatograms (EICs) of targeted analytes in authentic standards. Figure S2: Correlation of urinary NAT and blood acetate levels in the animal models of hyperacetatemia.
