# Supplementary material for: Characterization of Urinary N-Acetyltaurine as a Biomarker of Hyperacetatemia in Mice
Source: Metabolites. 2024 Jun 7;14(6):322. doi: 10.3390/metabo14060322 (PMC11205699; doi:10.3390/metabo14060322)

Characterization of Urinary *N*-Acetyltaurine as a Biomarker of Hyperacetatemia in Mice

Qingqing Mao[1], Xiaolei Shi[1], Yiwei Ma[1], Yuwei Lu[1], and Chi Chen[1,*]

[1]Department of Food Science and Nutrition, University of Minnesota, 1334 Eckles Ave., St. Paul, MN 55108, USA; maoxx113@umn.edu (Q.M.); shixx121@umn.edu (X.S.); maxxx792@umn.edu (Y.M.); luxxx572@umn.edu (Y. L.); chichen@umn.edu (C.C.)

*Correspondence: chichen@umn.edu. Tel.: +1-612-624-7704; Fax: +1-612-625-5272.

SUPPLEMENTARY DATA

Table S1. Reagents and chemicals used in sample preparation and LC-MS analysis.

Chemical/Reagents	Vendor
Acetic acid, Acetone (LC-MS grade), Acetonitrile (LC-MS grade, ACN), Ammonium formate, Formic acid (LC-MS grade), Water (LC-MS grade)	Fisher Scientific (Houston, TX)
2-Hydrazinoquinoline (HQ), Triphenylphosphine (TPP)	Alfa Aesar (Ward Hill, MA)
N-acetyl-L-glutamine, Ammonium carbonate ($(NH_4)_2CO_3$), 2-2′-Dipyridyl disulfide (DPDS)	MP Biomedicals, LLC (Irvine, CA)
Creatinine, D_4-acetic acid, Dansyl chloride (DC), Leucine enkephalin, Sodium carbonate (Na_2CO_3),	Sigma-Aldrich (St. Louis, MO)
D_5-Tryptophan	Cambridge Isotope Laboratories (Tewksbury, MA)
Taurine	Thermo Fisher Scientific (Waltham, MA)
Acetyl-L-glutamic acid, Acetyl-L-isoleucine, Acetyl-L-leucine, Acetyl-L-phenylalanine, Acetyl-L-tyrosine	Chem-Impex International (Wood Dale, IL)
Ethanol (reagent alcohol)	Ricca Chemicals (Arlington, TX)

Table S2. LC-MS instrumental settings for data acquisition.

Target compounds	LC column and temperature	LC mobile phase	Lock mass and MS detection mode	Capillary and cone voltage	Source and desolvation temperature	Cone and desolvation gas flow	*Collision gas and energy ramp
Taurine (DC derivatization), and *N*-acetyl amino acids (except for NAT)	Waters BEH C18 (reverse phase), 40 °C	A: 0.1% formic acid in water<br>B: 0.1% formic acid in ACN	Leucine enkephalin ($[M + H]^+$ = m/z 556.2771), Positive	0.2 kV, 40 V	120 °C, 350 °C	50 L/hr, 600 L/hr (N_2)	Argon, 10-50 eV
Acetic acid (HQ derivatization)	Waters BEH C18 (reverse phase), 40 °C	A: 2 mM NH_4OAc in water with 0.05% acetic acid<br>B: 2 mM NH_4OAc in 95% ACN and 5% water with	Leucine enkephalin ($[M + H]^+$ = m/z 556.2771), Positive	0.2 kV, 40 V	120 °C, 350 °C	50 L/hr, 600 L/hr (N_2)	NA

		0.05% acetic acid					
NAT	Waters BEH C18 (reverse phase), 40 °C	A: 0.1% formic acid in water B: 0.1% formic acid in ACN	Leucine enkephalin ([M - H]$^-$ = m/z 554.2615), Negative	-0.2 kV, -40 V	120 °C, 350 °C	50 L/hr, 600 L/hr (N_2)	NA
Creatinine	Waters Amide (HILIC), 40 °C	A: 0.1% formic acid in water B: 0.1% formic acid in ACN	Leucine enkephalin ([M + H]$^+$ = m/z 556.2771), Positive	0.2 kV, 40 V	120 °C, 350 °C	50 L/hr, 600 L/hr (N_2)	NA

**Settings for MSMS fragmentation analysis. NA: not applicable.*

Table S3. Information on selected triacetin-responsive urinary metabolites. Enlisted metabolites are selected from the volcano plot of the mouse urine metabolome with greater fold change (FC) and smaller *p*-values than NAT (Figure 1M). Accurate mass-based elemental composition analysis was performed to determine potential molecular formula.

Retention time (min)	Detected M/Z	Ion adduct	FC#	*p*-value	Potential molecular formula	Monoisotopic M/Z	Mass deviation (Δppm)
5.9152	240.232	[M+H]$^+$	7.7838	1.36E-06	$C_{15}H_{29}NO$^	239.2249	3
2.11	391.0629	[M+H]$^+$	7.763	2.73E-06	$C_{14}H_{10}N_6O_8$^	390.056	2
6.1748	459.4871	[M+H]$^+$	8.0529	5.46E-06	$C_{28}H_{62}N_2O_2$^	458.4811	4
2.1065	177.0741	[M+H]$^+$	8.188	5.58E-06	$C_7H_{12}O_5$^	176.0685	9
6.2401	485.1465	[M-H]$^-$	8.5178	5.75E-06	$C_{26}H_{22}N_4O_6$^	486.1539	2
2.1269	353.1073	[M+H]$^+$	8.4198	6.22E-06	$C_{23}H_{14}NO_3$^	352.0974	7
6.6641	515.5503	[M+H]$^+$	7.7516	7.91E-06	$C_{32}H_{70}N_2O_2$^	514.5437	3
2.1197	370.1341	[M+H]$^+$	9.7848	1.22E-05	$C_{14}H_{27}NO_6S_2$^	370.1353	3
1.0254	309.0815	[M-H]$^-$	8.6626	1.31E-05	$C_{11}H_{18}O_{10}$^	310.0907	5
3.1667	229.0163	[M-H]$^-$	8.7126	2.46E-05	$C_9H_{10}O_5S$^	230.0249	6
0.3157	166.0169	[M-H]$^-$	7.6126	9.07E-05	$C_4H_9NO_4S$*	167.0252	6

#FC, fold change, calculated by the relative abundance of metabolites in the triacetin group divided by that of the glycerol group;
^ Database seaech yielded no match with known urinary metabolites
**N-acetyltaurine, identity confirmed by comparison with authentic standard*

Figure S1. Extracted ion chromatograms (EICs) of targeted analytes. The retention time (RT), and monoisotopic mass-over-charge ratio (M/Z) of the detected ion adducts of analysts are enlisted. A) Creatinine, RT = 2.56, $[M+H]^+$ = 114.0667. B) Acetic acid, RT = 2.32 min, $[M+HQ]^+$ = 202.0980. C) NAT, RT = 0.33 min, $[M-H]^-$ = 166.0174. D) Taurine, RT = 3.4 min, $[M+DC]^+$ = 359.0736. E) *N*-acetyl-glutamine (NA-Gln), RT = 0.35 min, $[M+H]^+$ = 189.0875. F) *N*-acetyl-glutamic acid (NA-Glu), RT = 0.6 min, $[M+H]^+$ = 190.0715. G) *N*-acetyl-tyrosine (NA-Tyr), RT = 2.1 min, $[M+H]^+$ = 224.0923. H) *N*-acetyl-phenylalanine (NA-Phe), RT = 3.44 min, $[M+H]^+$ = 208.0974. I) *N*-acetyl-leucine (NA-Leu), RT = 2.97 min; *N*-acetyl-isoleucine (NA-Ile), RT = 3.13 min, $[M+H]^+$ = 174.1130. J) d_4-Acetic acid, RT = 2.32 min, $[M+HQ]^+$ = 205.1169. K) d_5-Tryptophan, RT = 5.71 min, $[M+DC]^+$ = 443.1801. L) Sulfadimethoxine (SDM), RT = 4.68 min in a BEH C18 column, $[M-H]^-$ = 309.0658. M) Sulfadimethoxine (SDM), RT = 0.68 min in an Amide column, $[M+H]^+$ = 311.0814.

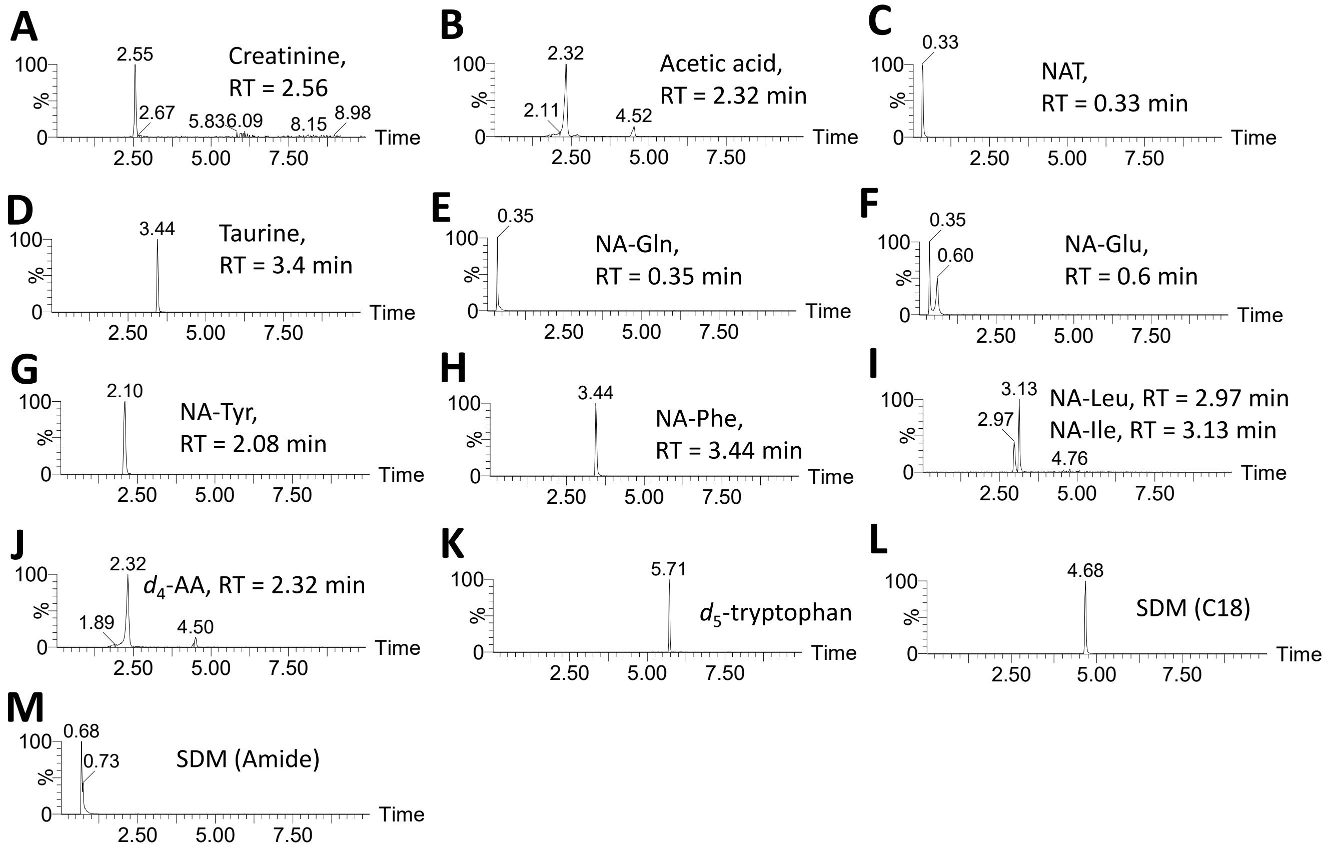

Figure S2. Correlations of urinary NAT and serum acetate levels in the animal models of hyperacetatemia. The correlations were examined by linear regression. A) The correlation in all three animal models, including triacetin-dosing, EtOH-dosing, and STZ-dosing with $R^2 = 0.55$ and *p*-value of the slope < 0.0001. B) The correlation in EtOH-dosing and STZ-dosing animal models, excluding triacetin-dosing, with $R^2 = 0.02$ and *p*-value of the slope = 0.4.

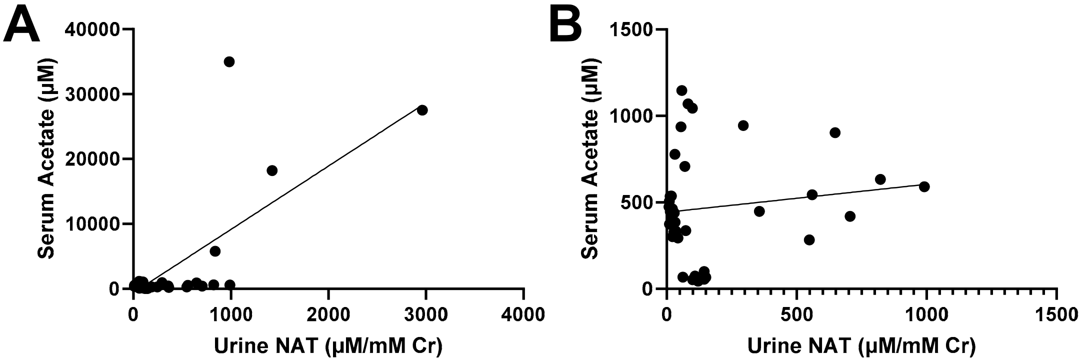

Supplement: Supplementary file 1 [file metabolites-14-00322-s001.zip › metabolites-3036792-supplementary.pdf]
